# Rosiglitazone Protects against Acetaminophen-Induced Acute Liver Injury by Inhibiting Multiple Endoplasmic Reticulum Stress Pathways

**DOI:** 10.1155/2022/6098592

**Published:** 2022-12-21

**Authors:** Yuping Cao, Wei He, Xiaoping Li, Jiahui Huang, Junxian Wang

**Affiliations:** ^1^College of Medicine, Anhui University of Science and Technology, Huainan 232001, China; ^2^Anhui No.2 Provincial People's Hospital, Hefei 230041, China

## Abstract

**Background:**

Excessive acetaminophen (APAP) use can lead to acute liver injury (ALI) by inducing endoplasmic reticulum stress (ERS). We previously found that pretreatment with the peroxisome proliferator-activated receptor-*γ* (PPAR-*γ*) ligand rosiglitazone (RSG) alleviated ALI in APAP-treated mice.

**Objective:**

To examine if RSG-mediated hepatoprotection is associated with ERS suppression.

**Methods:**

Forty-eight male CD-1 mice were randomly divided into control, RSG, APAP 4 h, APAP 24 h, RSG + APAP 4 h, and RSG + APAP 24 h groups. The RSG and RSG + APAP groups received RSG (20 mg/kg) by gavage 48, 24, and 1 h before intraperitoneal injection of 300 mg/kg APAP, while the APAP group received APAP alone and the control group received only normal saline. Animals were sacrificed immediately (RSG and control groups), 4 h (APAP 4 h and RSG + APAP 4 h), or 24 h (APAP 24 h and RSG + APAP 24 h) post-APAP injection. Liver tissues were collected for hematoxylin-eosin staining, TUNEL staining, and Western blotting for ERS-associated proteins. Serum alanine aminotransferase (ALT) and aspartate aminotransferase (AST) levels were also measured. A second cohort received APAP or RSG + APAP as described and were monitored for survival over one week.

**Results:**

At 4 and 24 h following APAP injection alone, serum ALT and AST levels were significantly elevated, and central lobular necrosis of the liver was observed. Necrosis area reached 21.7% at 4 h and 32.1% at 24 h post-APAP, while apoptotic fractions reached 25.6% and 32.4%. Further, 50% of mice in the survival analysis cohort died within one week post-APAP. At 4 h post-APAP, the ERS marker glucose-regulated protein-78 (GRP78) and ERS-associated proteins pJNK, GRP78, p-eIF2*α*, pPERK, and pIRE were all significantly upregulated. Pretreatment with RSG significantly reduced serum ALT and AST, liver necrosis area, apoptosis rate, and expression of ERS-associated proteins compared to APAP alone, while increasing survival to 80%.

**Conclusions:**

Rosiglitazone pretreatment can alleviate APAP-induced ALI by suppressing three branches of ERS signaling.

## 1. Introduction

Liver is the largest metabolic organ in the human body. Drugs, toxins, and various other chemicals are metabolized by the liver and ultimately excreted. Certain drugs or metabolites may interfere with hepatocellular metabolism or induce oxidative stress, leading to drug-induced liver injury (DILI) [1]. In addition to disrupting normal liver function, DILI can induce hypersensitivity or reduce tolerance to drugs during clinical treatment. In severe cases, DILI may even endanger patient lives [2].

Acetaminophen (APAP) is a nonsteroidal anti-inflammatory drug (NSAID) frequently recommended or prescribed in clinical practice, but excessive use (>10 g/day) can lead to liver injury and even death [3]. In the U.S., 26000 patients with APAP-associated ALI are hospitalized yearly and approximately 500 of them die from APAP-associated ALI. Therefore, it is of clinical significance to unravel the underlying mechanism and explore prevention and treatment strategies for APAP-associated ALI [4]. Although the precise mechanisms remain elusive, inflammatory reactions [5], oxidative stress [6], mitochondrial autophagy [7], and endoplasmic reticulum stress (ERS) [8] are implicated.

The endoplasmic reticulum (ER) provides a specialized intracellular environment for protein processing, folding, and sorting for intra- and extracellular transport. Endoplasmic reticulum stress (ERS) results when ER structures are damaged or the synthesis of proteins that require processing and packaging exceeds the functional limit of ER [9]. Transient ERS helps protect normal cellular function under stress. However, persistent ERS may lead to cell apoptosis, thereby impairing organ function [10]. Persistent ERS can also increase the production of reactive oxygen species (ROS), thus aggravating oxidative stress [11], or induce inflammatory reactions, further aggravating cell damage [12].

Peroxisome proliferator-activated receptors (PPARs) are members of the nuclear hormone receptor superfamily that upon ligand activation bind to DNA and regulate the transcription of downstream genes. The PPAR-*γ* isoform is a major regulator of gene expression in liver under both physiological and pathophysiological conditions [13], including in response to DILI [14], although the precise mechanisms are unclear. We recently reported that the PPAR ligand RSG suppressed APAP-induced acute liver injury, possibly by downregulating APAP-induced nicotinamide adenine dinucleotide phosphate oxidase (NOX) activity [15], thereby mitigating oxidative stress. However, we have not further elucidated the downstream mechanism of oxidative stress during APAP-induced ALI. Acute liver injury induced by APAP is also associated with ERS [7], and PPAR-*γ* signaling pathways can mitigate organ injury mediated by ERS [12], suggesting that PPAR-*γ* signaling may also prevent APAP-induced acute liver injury but modulating ER signaling pathways and suppressing ERS.

The current study was designed to evaluate the potential protective efficacy of RSG against APAP-induced acute liver injury and the contributions of ERS modulation, thereby providing experimental evidence for possible clinical DILI treatment.

## 2. Materials and Methods

### 2.1. Materials

#### 2.1.1. Laboratory Animals

Forty-eight 8-week-old male CD-1 mice weighing 28–30 g were purchased from Vital River Laboratory Animal Technology (Beijing, China. Permit Number: SCXK 2016-0006). All animals were fed adaptively for one week before drug administration. Animals were housed in an environment with control temperature (20–25°C) and humidity (50% ± 5%) under a 12 h light/12 h dark cycle with ad libitum access to food and water. All animal procedures were approved by the Research Ethics Committee of Anhui No.2 Provincial People's Hospital (2018-009).

#### 2.1.2. Drugs and Reagents

Rosiglitazone was purchased from Chengdu Hengrui Pharmaceutical (H20030569). Reagents for measuring reduced glutathione (GSH) in liver tissue as well as serum alanine aminotransferase (ALT) and aspartate aminotransferase (AST) detection kits were purchased from Nanjing Jiancheng Bioengineering Institute (Nanjing, China), acetaminophen from Sigma (St. Louis, MO, USA), nucleoprotein extraction kits and ECL detection kits from Pierce (Rockford, IL, USA) and primary antibodies against JNK, p-JNK, GRP78, pIRE1*α*, p-eIF2*α*, CHOP, Caspase 3, P50, ATF6, AKT, pAKT, *α*-tubulin, XBP1, and LaminA/C from Santa Cruz Biotechnology (Dallas, TX, USA). Protein markers were purchased from MBI Fermentas. Other reagents, if not specified, were also purchased from Sigma (USA).

### 2.2. Establishment of the APAP Mouse Model

Animals were randomly divided into six groups of 8: control (CON), RSG, APAP 4 h, APAP 24 h, RSG + APAP 4 h, and RSG + APAP 24 h. Mice in the APAP group were administered a single intraperitoneal injection of 300 mg/kg APAP as previously described [14], while mice in the RSG + APAP groups received 20 mg RSG by gavage (RSG dissolved in PBS solution) 48, 24, and 1 h before APAP administration and CON group mice received an equivalent volume of PBS by gavage. Finally, RSG group mice received 20 mg RSG by gavage at 48, 24, and 1 h, and then intraperitoneal injection of normal saline. Mice were sacrificed immediately post-APAP (0 h, CON and RSG groups), 4 h post-APAP (APAP 4 h and RSG + APAP 4 h groups), or 24 h post-APAP (APAP 24 h and RSG + APAP 24 h groups). All mice were weighed before sacrifice and blood samples were then collected for ALT and AST measurements. The whole liver was removed, weighed for liver weight/body mass index calculation, and cut into tissue segments. Tissue segments were fixed with 4% paraformaldehyde, embedded in paraffin, and sectioned for hematoxylin-eosin (HE) staining, liver cell apoptosis detection, and immunohistochemical staining. The remaining liver tissues were stored at -80°C for protein expression measurements.

### 2.3. Survival Rate of APAP-Treated Mice

To examine the effect of RSG on the survival rate of mice with APAP-induced acute liver injury, 20 healthy male CD-1 mice were randomly divided into equal two groups, one receiving a single intraperitoneal injection of APAP (300 mg/kg) alone and the other receiving 20 mg/kg RSG by gavage 48, 24, and 1 h prior to APAP. All mice in both groups resumed regular feeding 8 h after administration. The number of mice surviving was monitored and survival curves constructed.

### 2.4. Detection of Serum ALT and AST

The serum levels of ALT and AST were measured using detection kits strictly according to the manufacturer's instructions. Results are expressed as mean ± standard error (^−^*x* ± SEM).

### 2.5. Measurement of Reduced Glutathione (GSH) in Liver Tissues

Measurements were conducted strictly according to the manufacturer's instructions and as mean ± standard error (^−^*x* ± SEM).

### 2.6. HE Staining

Pieces of liver tissue were fixed in 4% paraformaldehyde for 24 h, dehydrated in gradient ethanol, cleared in xylene, embedded in paraffin, sectioned, stained with HE, and sealed with neutral gum. Pathological changes were photographed under light microscopy and the necrotic area calculated using ImageJ (NIH, Bethesda, MD, USA).

### 2.7. Extraction of Liver Total Proteins and Nucleoproteins for Western Blotting

Total protein and nucleoprotein were extracted strictly according to the kit manufacturer's instructions. The extracted protein solutions were quantified by the Lorry method and then adjusted to the same concentration prior to denaturation by bathing in boiling water for 10 min and storage at -20°C until analysis. Total liver protein and nucleoproteins were separated by SDS-PAGE electrophoresis and transferred onto polyvinylidene fluoride membranes. Membranes blotted with total protein were incubated with X primary antibodies against JNK, p-JNK, GRP78, pIRE1*α*, p-eIF2*α*, CHOP, Caspase-3, ATF6, AKT, pAKT, and *α*-tubulin (gel loading control) for 2 h, while membranes blotted with nucleoprotein extracts were incubated with *X* antibodies against XBP-1, P50, ATF6, and LaminA/C (gel loading control) for 2 h. Membranes were washed four times in DPBS containing 0.05% Tween-20 (10 min per wash), incubated with goat anti-rabbit and goat anti-mouse secondary antibodies for 2 h, then washed four times in DPBS containing 0.05% Tween-20 (10 min per wash). Target bands were visualized using an ECL detection kit.

### 2.8. TUNEL Staining

TUNEL staining was conducted to quantify the apoptosis rate of liver cells due to APAP and potential cytoprotection by RSG pretreatment using an *in situ* apoptosis detection kit according to the manufacturer's protocol. Sections were then counterstained with hematoxylin to visualize surviving cells. The number of TUNEL-positive cells was counted in 12 randomly chosen fields of 6 sections from 6 individual mice under 200× magnification and expressed as the percentage of all hematoxylin- and TUNEL-stained cells.

### 2.9. Statistical Analysis

All data are expressed as mean ± standard error (^−^*x* ± SEM). SPSS 17.0 statistical software was used for all statistical analyses. Treatment group means were compared by analysis of variance (ANOVA) with post hoc *X* tests for pair-wise comparisons. A *P* < 0.05 was considered significant for all tests.

## 3. Results

### 3.1. RSG Pretreatment Reduced APAP-Induced ALI and Mortality

**Figure 1:**
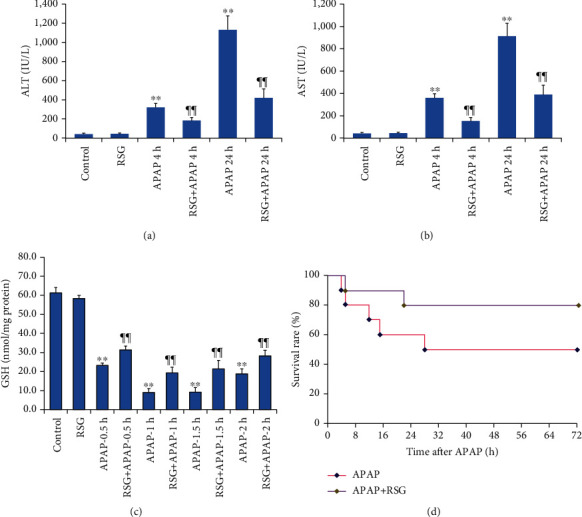
Pretreatment with rosiglitazone (RSG) prevented APAP-induced acute liver injury and reduced mortality. (a, b) Pretreatment with RSG reversed APAP-induced elevations in serum ALT (a) and AST (b). (c) Pretreatment with RSG prevented APAP-induced depletion of hepatocellular GSH. All data are expressed as mean ± SE (*n* = 8). ^∗∗^*P* < 0.01 compared to the control group (0 time point), ¶¶ *P* < 0.01 compared to the APAP group at the same time point. (d) Rosiglitazone pretreatment reduced APAP-induced mortality.

In the present study, we examined the hepatoprotective efficacy of RSG pretreatment against excessive APAP and the potential contribution of ERS suppression. Serum concentrations of ALT and AST were persistently elevated from 4 to 24 h after intraperitoneal injection of APAP alone (without RSG pretreatment), consistent with physiologically significant liver damage (Figures 1(a) and 1(b)), while RSG pretreatment significantly reversed these effects.

It is widely accepted that the major cause of APAP-induced ALI is depletion of reduced glutathione (GSH) in liver tissues and ensuing oxidative stress. Consistent with a contribution of oxidative stress to APAP-induced hepatotoxicity. A large quantity of GSH will be rapidly depleted after APAP-induced ALI. To evaluate the changing pattern of GSH depletion, the GSH contents in mouse liver tissues were measured at 0.5, 1, 1.5, and 2 h after APAP-induced ALI, respectively. The results showed that the GSH level was rapidly depleted at 0.5 h post-APAP injection, almost completely depleted at 1 h post-APAP injection, and the depletion status remained at 1.5 h post-APAP injection, whereas the GSH level in the liver tissues began to increase at 2 h after APAP injection. However, the GSH contents in the liver tissues of APAP-induced ALI mice pretreated by RSG were significantly higher than those in the APAP group at the same time points, respectively, (Figure 1(c)). In addition to reversing signs of liver pathology, RSG pretreatment also increased mouse survival within one week following APAP injection from 5 of 10 to 8 of 10 (Figure 1(d)).

Both absolute and relative liver weights were increased at 4 h after APAP injection, and were greater still at 24 h after APAP injection (Table 1), suggesting that ALT and AST release, GSH depletion, and mortality, liver hypertrophy were reversed by RSG pretreatment (Table 1).

### 3.2. RSG Pretreatment Inhibited APAP-Induced Liver Cell Necrosis and Apoptosis

**Figure 2:**
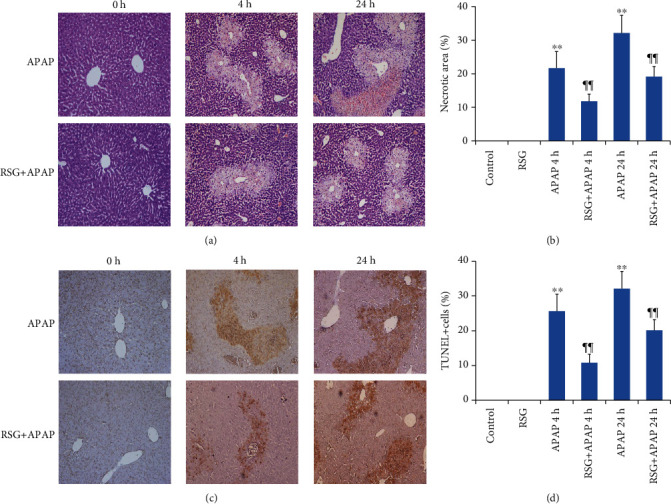
Rosiglitazone pretreatment mitigated APAP-induced hepatic pan-necrosis and apoptosis. (a) Hematoxylin and eosin staining of liver sections (magnification: 100×) showing regions of pan-necrosis. (b) Quantification of necrotic area. The necrotic areas in APAP-treated mice were significantly reduced by RSG pretreatment. (c) TUNEL staining of apoptotic hepatocytes (magnification: 100×). (d) RSG pretreatment significantly reduced the number of apoptotic (TUNEL+) hepatocytes in APAP-injected mice. All data are expressed as mean ± SE (*n* = 8). ^∗∗^*P* < 0.01 compared to the control group (0 time point), ¶¶ *P* < 0.01 compared to the APAP group at the same time point.

Histopathological staining of liver tissues from mice injected with APAP alone revealed typical central lobular necrosis (Figures 2(a) and 2(b)) that covered approximately 21.7% of liver section area 4 h post-APAP injection and 32.1% at 24 h post-injection. Consistent with previous signs of hepatoprotection, pretreatment with RSG significantly reduced liver necrosis area to 11.8% at 4 h and 19.1% at 24 h after APAP injection (Figures 2(a) and 2(b)).

TUNEL staining was adopted to determine the liver cell apoptosis rate due to APAP-induced ALI and possible reversal by RSG treatment. The number of TUNEL^+^ cells reached 25.6% at 4 h after APAP injection, and increased further to 32.4% at 24 h after APAP injection (Figures 2(c) and 2(d)), while RSG pretreatment significantly reduced apoptosis rates to 10.8% at 4 h and 20.1% at 24 host-APAP (Figures 2(c) and 2(d)).

### 3.3. RSG Pretreatment Inhibited APAP-Induced Activation of Endoplasmic Reticulum Stress

**Figure 3:**
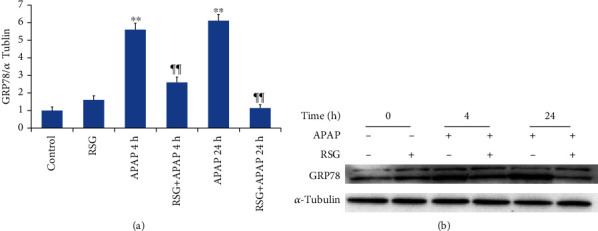
Pretreatment with RSG reduced APAP-induced hepatic upregulation of the ERS marker GRP78. (a) Hepatic p-GRP78 expression was measured by immunoblotting. Representative immunoblots of GRP78 (upper panel) and *α*-tubulin (lower panel) as the gel loading control are shown. (b) Densitometry of GRP78 expression. All data shown as mean ± SE (*n* = 8). ^∗∗^*P* < 0.01 compared to the control group (0 time point), ¶¶*P* < 0.01 compared to the APAP group at the same time point.

Treatment with APAP alone significantly upregulated hepatic expression of the ERS marker protein GRP78 at 4 h and further at 24 h posttreatment as detected by Western blotting, while RSG pretreatment significantly reduced this elevation at both times (Figure 3). These results suggest that ERS contributes to APAP-induced ALI and that RSG protects against ALI in part by mitigating ERS.

#### 3.3.1. RSG Pretreatment Inhibited the PERK-eIF2*α*-CHOP ERS Signaling Pathway in APAP-Induced ALI Model Mice

**Figure 4:**
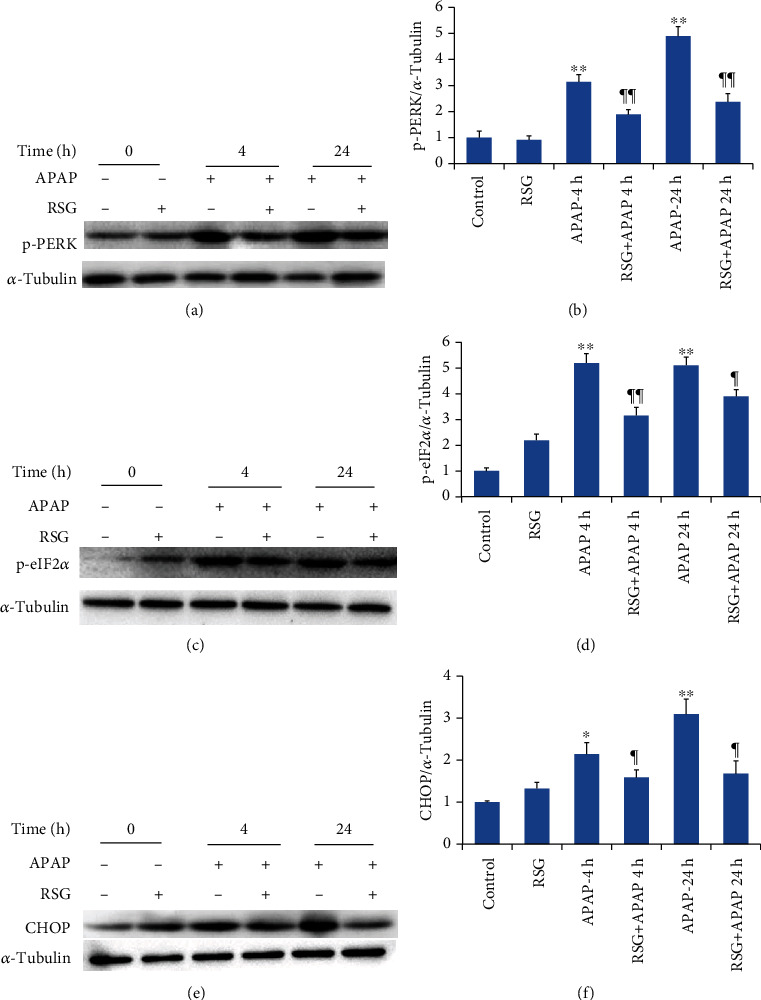
Pretreatment with RSG reversed APAP-induced activation of the hepatic PERK-eIF2*α*-CHOP pathway. (a) Representative immunoblots of hepatic p-PERK (upper panel) and *α*-tubulin (lower panel) as the gel loading control. (b) Densitometry of p-PERK expression showing upregulation by APAP and reversal by RSG pretreatment. (c) Representative immunoblots of hepatic p-eIF2 *α* (upper panel) and *α*-tubulin (lower panel). (d) Densitometry showing p-eIF2 *α* upregulation by APAP and reversal by RSG pretreatment. (e) Representative immunoblots of hepatic CHOP (upper panel) and *α*-tubulin (lower panel). (f) Densitometry showing CHOP upregulation by APAP and reversal by RSG pretreatment. All data are expressed as mean ± SE (*n* = 8). ^∗^*P* < 0.05 and ^∗∗^*P* < 0.01 compared to the control group (0 time point), ¶ *P* < 0.05 and ¶¶ *P* < 0.01 compared to the APAP group at the same time point.

Western blotting of liver tissue from APAP-induced ALI model mice also revealed increased phosphorylation levels of PERK and eIF2*α* proteins at 4 h and 24 h after APAP treatment compared to the CON group (Figures 4(a) and 4(b)). Again, RSG pretreatment partially reversed these changes (Figures 4(a) and 4(b)). Similarly, the expression level of CHOP was significantly elevated by APAP compared to the CON group, and this elevation was significantly reduced by RSG pretreatment compared to APAP treatment alone (Figure 4(c)). Thus, RSG appears to suppress PERK-eIF2*α*-CHOP ERS signaling pathway activation.

#### 3.3.2. RSG Pretreatment Inhibited the IRE-XBP1-JNK Signaling Pathway in APAP-Induced ALI Model Mice

**Figure 5:**
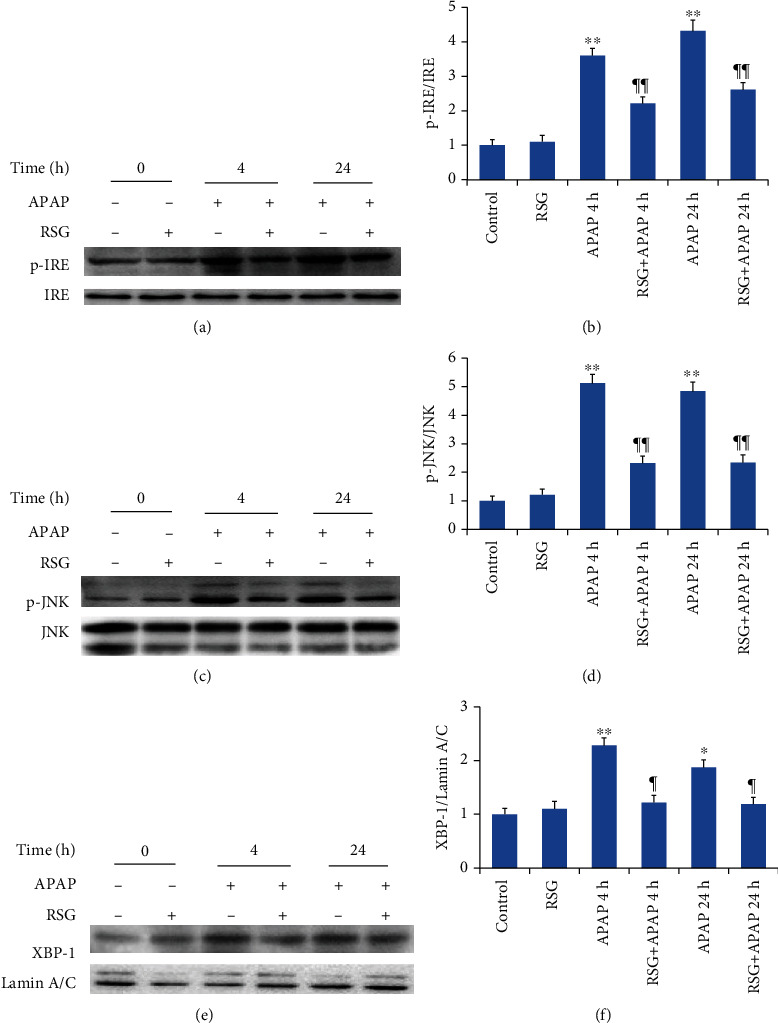
Rosiglitazone pretreatment suppressed APAP-induced activation of the hepatic IRE-XBP1-JNK pathway. (a) Representative immunoblots of hepatic p-IRE (upper panel) and IRE (lower panel). (b) Densitometry of p-IRE showing upregulation by APAP and reversal by RSG pretreatment. (c) Representative immunoblots of hepatic p-JNK (upper panel) and JNK (lower panel). (d) Densitometry of p-JNK showing upregulation by APAP and reversal by RSG pretreatment. (e) Representative immunoblots of hepatic XBP1 (upper panel) and LaminA/C (lower panel) as the gel loading control. (f) Densitometry of XBP1 showing upregulation by APAP and reversal by RSF pretreatment. All data are expressed as mean ± SE (*n* = 8). ^∗^*P* < 0.05 and ^∗∗^*P* < 0.01 compared to the control group (0 time point), ¶ *P* < 0.05 and ¶¶ *P* < 0.01 compared to the APAP group at the same time point.

Expression levels of phosphorylated IRE and JNK were also significantly elevated both 4 h and 24 h after APAP injection compared to vehicle-injected control mice (Figures 5(a) and 5(b)), and similar to PERK-eIF2*α*-CHOP ERS pathway components, upregulated expression levels of these IRE-XBP1-JNK signaling pathway components were reversed by RSG pretreatment (Figures 5(a) and 5(b)). In addition, the expression level of XBP1 in liver cell nuclei was significantly upregulated in APAP-induced ALI mice (Figure 5(c)), and this response was also reversed by RSG pretreatment.

#### 3.3.3. RSG Pretreatment Inhibited the ATF6 Signaling Pathway in APAP-Induced ALI Model Mice

**Figure 6:**
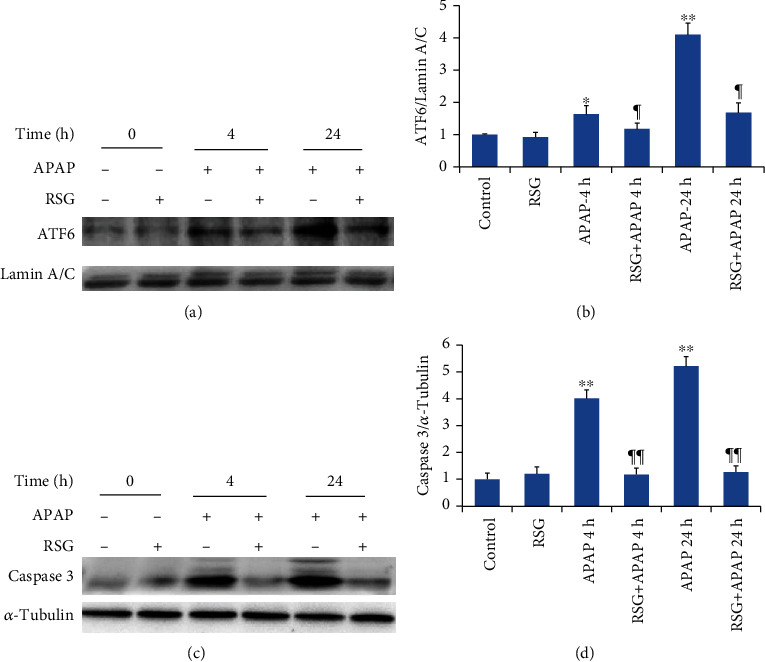
Rosiglitazone pretreatment suppressed APAP-induced activation of the hepatic ATF6 pathway. (a) Representative immunoblots of hepatic ATF6 (upper panel) and LaminA/C (lower panel). (b) Densitometry of ATF6 showing upregulation by APAP and reversal by RSG pretreatment. (c) Representative immunoblots of hepatic caspase-3 (upper panel) and *α*-tubulin (lower panel). (d) Densitometry of caspase-3 showing upregulation by APAP and reversal by RSG pretreatment. All data are expressed as mean ± SE (*n* = 8). ^∗^*P* < 0.05 and ^∗∗^*P* < 0.01 compared to the control group (0 time point), ¶ *P* < 0.05 and ¶¶ *P* < 0.01 compared to the APAP group at the same time point.

The expression level of ATF6 in liver cell nuclei was significantly higher 4 h and 24 h after APAP treatment compared to the CON group (Figure 6(a)) and this response as well was reversed by RSG pretreatment (Figure 6(a)). In addition, expression of the apoptosis effector caspase-3 was significantly upregulated in liver tissues of APAP-induced ALI mice and suppressed by RSF pretreatment (Figure 6(b)), consistent with TUNEL staining results.

#### 3.3.4. RSG Pretreatment Inhibited Activation of NF-*κ*B and PI3K/AKT Signaling Pathways in APAP-Induced ALI Model Mice

**Figure 7:**
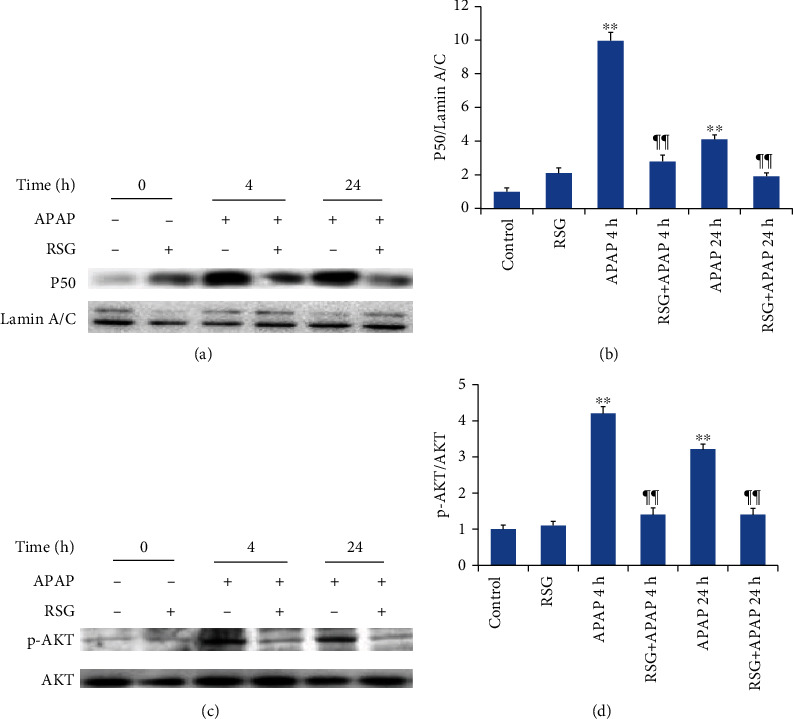
Rosiglitazone pretreatment suppressed APAP-induced activation of NF-*κ*B and PI3K/AKT pathways. (a) Representative immunoblots of NF-*κ*B (upper panel) and LaminA/C (lower panel). (b) Densitometry of NF-*κ*B showing upregulation by APAP and reversal by RSG pretreatment. (c) Representative immunoblots of p-AKT (upper panel) and AKT (lower panel). (d) Densitometry of p-AKT showing upregulation by APAP and reversal by RSG pretreatment. All data are expressed as mean ± SE (*n* = 8). ^∗∗^*P* < 0.01 compared to the control group (0 time point). ¶¶ *P* < 0.01 compared to the APAP group at the same time point.

Finally, expression of the stress-associated transcription factor NF-*κ*B p50 was upregulated in liver cell nuclei 4 h and 24 h after APAP injection (Figure 7(a)), and this upregulation was reversed by RSG pretreatment (Figure 7(a)). Similarly, the PI3K/AKT inflammatory signaling pathway component pAKT was upregulated at 4 h and 24 h after APAP injection (Figure 7(b)), and this response was attenuated by RSG pretreatment (Figure 7(b)).

## 4. Discussion

We demonstrate that pretreatment with the PPAR-*γ* agonist rosiglitazone can mitigate acute liver injury in mice caused by excessive acetaminophen intake. Rosiglitazone pretreatment significantly reduced APAP-induced elevations of serum ALT and AST, two common clinical signs of liver damage. In accord with this observation, RSG reduced liver pannecrosis area and apoptosis rate. Notably, these effects were associated with lower short-term mortality. Pretreatment with RSG also reduced APAP-induced upregulation of multiple ERS markers and signaling factors, suggesting that the protective effects of RSG pretreatment are mediated at least in part by inhibition of ERS in the liver.

The main pathological manifestations of APAP-induced ALI include necrosis and apoptosis of liver cells. Liver cell necrosis causes the release of liver-specific enzymes ALT and AST into the blood. In addition, previous research suggests that Caspase activation and liver cell apoptosis do not occur during APAP-induced ALI [16]. Nevertheless, TUNEL staining has been employed to evaluate the severity of APAP-induced ALI in more studies [17]. Hence, in the present study, we still adopted TUNEL staining to assess the degree of APAP-induced ALI. TUNEL staining revealed that the number of dead liver cells was significantly increased at 4 h post-APAP injection and was even higher at 24 h post-APAP injection, suggesting induction of a progressive degenerative process. Notably, RSG significantly reduced the number of dead cells at both 4 and 24 h post-APAP and ultimately reduced mortality, suggesting that RSG inhibited this degenerative process, and further observations indicated that this protection stemmed from ongoing suppression of ERS.

Numerous studies have implicated ERS liver diseases, including fatty liver [18], viral hepatitis [19], ischemia-reperfusion injury [20], liver fibrosis [21], and drug-induced liver injury [22]. Further, suppressing ERS can prevent the progression of liver diseases [23]. The expression level of the ERS marker protein GRP78 [24] was significantly elevated at both 4 and 24 h following APAP injection, while RSG pretreatment significantly reduced hepatic GRP78 expression at the same time points, further supporting suppression of ERS as an important mechanism mediating protection against APAP-induced ALI by RSG.

Endoplasmic reticulum stress is associated with the induction of multiple homeostatic signaling pathways that may become pathogenic. Previous studies have reported that the PERK-eIF2*α*-CHOP signaling pathway is involved in the progression of nonalcoholic fatty liver disease, as well as in liver cell apoptosis and autophagy [25]. Moreover, inhibiting activation of the PERK-eIF2*α*-CHOP signaling pathway can alleviate drug-induced liver injury [26]. We found that the PERK-eIF2*α*-CHOP signaling pathway was activated by APAP as evidenced by elevated phosphorylation of PERK and eIF2*α* proteins and upregulated expression of CHOP at 4 to 24 h after APAP injection. In contrast, RSG pretreatment reduced the expression level of p-PERK, p-eIF2*α*, and CHOP at both time points. Therefore, we speculate that the PERK-eIF2*α*-CHOP signaling pathway contributes to the progression of APAP-induced ALI and that RSG pretreatment protects against ALI in part by suppressing PERK-eIF2*α*-CHOP signaling.

Inositol-requiring enzyme 1 (IRE1) is a transmembrane protein located on the ER membrane. In response to ERS, IRE1 dissociates from GRP78 to form a homodimer, leading to its autophosphorylation and activation. Activated IRE1*α* has endonuclease activity that can splice X-box binding protein 1 (XBP-1) mRNA to form a new transcript encoding the active transcription factor spliced XBP1 (XBP1s), which in turn is transferred to the nucleus where it can upregulate the expression levels of ERS-associated molecular chaperones, folding enzymes, and other related genes. In addition, IRE1*α* can reduce the transcription of mRNAs encoding secreted and transmembrane proteins, thereby easing the protein load in the ER [27]. Further, IRE1*α*-regulated signals such as tumor necrosis factor receptor-associated factor-2 (TRAF2) and signal-regulated kinase 1 (ASK1)/c-Jun N-terminal kinase (JNK) can promote autophagy, insulin resistance, and apoptosis [28]. Western blotting revealed increased p-IRE1*α* expression in the liver nuclear protein fraction at 4 h and 24 h after APAP treatment. Expression of XBP1 was also significantly upregulated in the nuclear protein fraction following APAP treatment, suggesting that the IRE1 branch of the ERS signaling pathway contributes to ALI and that pathway suppression contributes to the protective efficacy of RSG. The c-Jun N-terminal kinase (JNK) is an important downstream effector of IRE signaling under ERS [29], and phosphorylated (activated) JNK is a major initiator of cell apoptosis [30]. In the present study, p-JNK expression was significantly increased at 4 h and 24 h after APAP treatment, and this elevation was reversed by RSG pretreatment.

ATF6 is an ER transmembrane protein involved in the unfolded protein response (UPR). In response to ERS, ATF6 dissociates from GRP78, and is transferred to Golgi apparatus, where it is spliced and activated by proteases. Activated ATF6 can then be transferred into the nucleus to form a homodimer or heterodimers with other transcription factors, including ATF4 and XBP1. These factors can activate the ERS reaction element of cis-acting element and upregulate the expression of molecular chaperones to enhance the folding and elimination of accumulated proteins. ATF6 is also a critical regulator of CHOP [31] and activation of the ATF6 signaling pathway is strongly associated with cell apoptosis [32]. In the present study, Western blotting revealed that ATF6 nucleoprotein was significantly upregulated in liver tissue at 4 h and 24 h after APAP treatment, suggesting that in the process of APAP-induced ALI in mice, the ATF6 branch of the UPR is activated after CCl4 induction. Notably, elevated expression was reversed by RSG, suggesting that RSG pretreatment may reduce activation of the ERS signaling pathway ATF6 branch.

There is accumulating evidence that PPAR-*γ* has anti-inflammatory activity [33]. For instance, it has been reported that PPAR-*γ* signaling protects against nonalcoholic steatohepatitis by suppressing inflammation [34]. A previous study reported that PPAR-*γ* agonists can inhibit the production of inflammatory cytokines in liver during APAP-induced injury [35]. We found that the stress-associated transcription factor NF-*κ*B was activated during APAP-induced ALI. The expression level of NF-*κ*B subunit p50 was significantly upregulated in liver cell nuclei at 4 h and 24 h after APAP injection. In addition, the expression level of phosphorylated AKT, a signaling molecule within the immune-modulating PI3K/AKT signaling pathway, was also significantly upregulated after APAP induction. Collectively, these findings suggest that excessive APAP induces an inflammatory reaction in the liver and that RSG pretreatment reduces ALI by acting to suppress NF-*κ*B and PI3K/AKT inflammatory signaling. Considering the intimate relationship between ERS and inflammation, inhibiting ERS can significantly alleviate inflammatory response [36]. Consequently, these findings suggest that RSG pretreatment may prevent APAP-induced ALI via PPAR-*γ*-mediated anti-ERS and anti-inflammatory signaling.

## 5. Study Limitations

This study has several limitations. First, we did not examine expression of PPAR-*γ* in the liver cell nucleus, so it is possible that a non-PPAR-*γ* signaling pathway is involved in the protective effect of RSG on APAP-induced ALI. Second, the precise mechanisms for PPAR-*γ*-mediated inhibition of ERS was not explored. In this study, although RSG pretreatment can mitigate oxidative stress and ERS in APAP-induced ALI, the causal relationship between oxidative stress and ERS has not be investigated, and validating this causal relationship is of significance. Therefore, additional studies are required to unravel the therapeutic mechanisms.

## 6. Conclusions

Pretreatment with RSG can prevent or reduce the severity of ALI induced by APAP in mice, potentially by suppressing three branches of the ERS signaling network in liver cells. These findings suggest that PPAR-*γ* serves as an important regulator of ERS in liver cells. Therefore, PPAR-*γ* agonists may be effective drugs to prevent the progression of APAP-induced ALI in clinical practice.

## Figures and Tables

**Table 1:** Effects of RSG pretreatment on absolute liver weight and liver/body weight.

	Control	RSG	APAP4h	RSG + APAP4h	APAP24h	RSG + APAP24h
Body weight (g)	30.88 ± 0.77	32.10 ± 057	32.30 ± 0.89	30.60 ± 0.60	31.92 ± 0.1	29.90 ± 0.49
Liver weight (g)	1.30 ± 0.07	1.37 ± 0.03	1.65 ± 0.03^∗∗^	1.34 ± 0.05¶	1.63 ± 0.11^∗∗^	1.33 ± 0.04¶
Liver/body weight ratio (%)	4.20 ± 0.18	4.25 ± 0.07	5.12 ± 0.10^∗∗^	4.35 ± 0.14¶	5.08 ± 0.21^∗∗^	4.44 ± 0.11¶

^∗∗^ *P* < 0.01 compared to the control group (0 time point). ¶ *P* < 0.05 compared to the APAP group at the same time point.

## Data Availability

All data included in this study are available upon request by contact with the corresponding author.
